# Enhanced thermal properties of poly(lactic acid)/MoS_2_/carbon nanotubes composites

**DOI:** 10.1038/s41598-020-57708-1

**Published:** 2020-01-20

**Authors:** Piotr Homa, Karolina Wenelska, Ewa Mijowska

**Affiliations:** West Pomeranian University of Technology, Szczecin, Faculty of Chemical Technology and Engineering, Nanomaterials Physicochemistry Department, Piastów Ave. 42, 71-065 Szczecin, Poland

**Keywords:** Chemical engineering, Nanocomposites

## Abstract

In this work, few-layered molybdenum disulfide (MoS_2_) was functionalized with metal oxide (M_x_O_y_) nanoparticles which served as a catalyst for carbon nanotubes (CNT) growth in the chemical vapour deposition (CVD) process. The resulting MoS_2_/M_x_O_y_/CNT functionalized nanomaterials were used for flame retarding application in poly(lactic acid) (PLA). The composites were extruded with a twin-screw extruder with different wt% loading of the nanomaterial. Full morphology characterization was performed, as well as detailed analysis of fire performance of the obtained composites in relation to pristine PLA and PLA containing an addition of the composites. The samples containing the addition of MoS_2_/M_x_O_y_/CNT displayed up to over 90% decrease in carbon oxide (CO) emission during pyrolysis in respect to pristine PLA. Microscale combustion calorimetry testing revealed reduction of key parameters in comparison to pristine PLA. Laser flash analysis revealed an increase in thermal conductivity of composite samples reaching up to 65% over pristine PLA. These results prove that few-layered 2D nanomaterials such as MoS_2_ functionalized with CNT can be effectively used as flame retardance of PLA.

## Introduction

Greater use of conventional petroleum based commodity resins in various applications have led to inevitable rise of its ecological footprint. Various new eco-friendly biodegradable polymers were developed in order to counteract this effect. With its biodegradability, low emission of greenhouse gas and low production energy PLA currently stands as one of popular alternatives for conventional polymer materials^1^. Due to its high degree of transparency, good mechanical properties, low toxicity and ability to process using equipment as well as relatively low cost and large production volume it shows high potential for packaging, household and biomedical applications. Nevertheless, other potential applications such as in electronics or automotive industry require PLA grades with high impact strength, better processing ability and improved flame retardant behaviour^2^. In order to meet that demand novel methods of modification need to be developed and applied.

A common way of improving flame retardancy of polymer is through combining its matrix with flame retardant filler. In the past improved flame resistance was achieved by introduction of halogenated flame retardants (HFR). Nowadays, it faces severe restrictions in Europe and North America due to the release of toxic substances and large amounts of smoke during combustion^3^. Instead, halogen-free flame retardant formulations function more commonly now as an environmentally friendly alternatives for HFR. These include intumescent flame retardants^4,5^, phosphorus- and nitrogen-containing micro- and nanoparticles^6–8^, inorganic substances and silica derivatives^9^ which can be used either individually or in combination^10^ to achieve optimal flame retardant performance.

Polymer/layered inorganic materials composites have high potential for improving thermal properties of polymers. Molybdenum disulfide is a member of a family of transition metal dichalcogenides (TDM). Structurally its crystals are characterized as hexagonal layered configurations. Atoms in the layer are bonded with strong covalent bonding, while layers are packed together to form a sandwich structure with weak Van der Waals forces similarly to graphite or boron nitride^11^. With their unique electrical, optical, thermal and mechanical properties MoS_2_ nanosheets can be potentially used for application as fillers for improving properties of polymers. Being a representative of layered inorganic materials MoS_2_ is expected to disperse and exfoliate in polymers, which results in the physical barrier effects that inhibits the diffusion of heat and gaseous decomposition products^12,13^. Molybdenum, a transition metal element, promotes the formation of charred layer during the combustion which acts as a physical barrier that slows down the heat and mass transfer^11^. In order to achieve high-performance homogenous dispersion of MoS_2_ nanosheets in the polymer hosts and proper interfacial interactions need to be established^14^. Similarly to metal oxide/graphene hybrid materials addition of metal oxide/MoS_2_ nanoparticles might prevent the aggregation of MoS_2_ flakes during preparation of polymer nanocomposites and result in improved flame retardant performance^15,16^. In addition they can also lead to improved char generation due to catalytic activity of metal oxide as well as suppressed smoke production and reduced toxicity due to catalytic conversion of carbon oxide (CO)^17^.

Another possible alternative to the use of conventional flame retardants are carbon nanotubes (CNT). These can be introduced into the polymer matrix in pristine form of a small diameter (1–2 nm) single-walled carbon nanotubes or a large diameter (10–100 nm) multi-walled carbon nanotubes (SWCNT and MWCNT, respectively)^18–23^. In addition to this functionalization of CNT can also be carried out in order to significantly improve the flame retardant performance^17^. This can be performed in three different ways. Surface modification by coupling agents allows for enhancement of dispersion state of CNT^24–26^. For example, addition of 9 wt% content of CNT functionalized with vinyltriethoxysilane into epoxy composite allowed for increase of limiting oxygen index (LOI) from 22 to 27% and improvement of UL-94 rating from V-1 to V-0^25^. This also resulted in increase of char yield at 750 °C by 46.94%. Char yield can be also enhanced through covalent linkage of organic flame retardants to CNT^27–30^ following surface treatment. Functionalization of CNT can facilitate their dispersion within the polymer matrix and enhance interfacial adhesion between the CNT and the polymer^30^. Formation of genuine composites can be confirmed by an increase in the Young’s modulus and flame resistance of compositions containing pristine CNT^28^. Finally, hybridization of CNT by inorganic particles can allow for superior flame retardant properties^31–33^. In addition to that other key parameters, such as thermal stability and dielectric properties, can be also enhanced^31^. Generally speaking, flame retardant actions of CNT/polymer composites involve the condensed phase action. Char layer formed on the entire sample surface acts as insulation layer that reduces the amount of volatiles escaping to the flame. The formation of continuous layer is obtained by formation of three-dimensional network structure when the content of CNT reaches a threshold value^17^. Good quality char plays major role in reduction of peak heat release rate (pHRR)^34,35^.

During the scope of presented research few-layered MoS_2_ nanosheets functionalized with M_x_O_y_ nanoparticles served as catalysts for growth of CNT in the CVD process were prepared. The obtained nanomaterials were used as flame retarding agents in PLA composites. Detailed description of synthesis, as well as full characteristics and analysis of properties of obtained PLA-based composites were provided. Additionally, a thorough analysis of thermal stability, fire performance and thermal conductivity of these materials was also performed and discussed in details.

## Methods

### Materials

Bulk MoS_2_ (powder), N-Methyl-2-pyrrolidone (NMP) (anhydrous, 99.5%), cobalt(II) acetate tetrahydrate (99%), iron(II) acetate (95%) and nickel(II) acetate tetrahydrate (98%) were purchased from Merck. PLA was obtained from Goodfellow. Hydrogen peroxide (30%) and ethanol (96%) were purchased from Chempur. Gaseous nitrogen and ethylene were purchased from Messer and Air Liquide, respectively.

### Preparation of few-layered MoS_2_

1 g of bulk MoS_2_ powder was transferred into a 250 mL flat-bottomed beaker filled with solution of 95 mL of NMP and 5 mL of hydrogen peroxide. This was followed by 30 minutes of continuous sonication in an ultrasonic washer, after which solution was transferred to a 250 mL round bottomed flask, plugged to reflux and consciously stirred at 360 rpm and 35 °C for 24 h. Final dispersion was centrifuged four times at 10000 rpm for 20 minutes and washed with ethanol.

### Preparation of MoS_2_/M_x_O_y_/CNT

In order to obtain MoS_2_/M_x_O_y_/CNT modified nanomaterials following procedure was applied. First, 150 mg of few-layered MoS_2_ and 150 mg of respective source of metal oxide (cobalt(II) acetate tetrahydrate, iron(II) acetate or nickel(II) acetate tetrahydrate) were dispersed in ethanol and sonicated in an ultrasonic washer for 2 h. Next, dispersions were stirred for 48 h, after which they were dried under high vacuum at 440 °C for 3 h. This resulted respectively in MoS_2_/Co_2_O_3_, MoS_2_/Fe_2_O_3_ and MoS_2_/Ni_2_O_3_ modified nanomaterials. Next CVD was performed in tube furnace under constant 100 mLmin^−1^ flow of nitrogen at 850 °C with a 15 minute long, 60 mLmin^−1^ flow of ethylene as a source of carbon.

### Extrusion of MoS_2_/M_x_O_y_/CNT modified PLA composites

PLA was utilized as a polymer matrix. Four composites were prepared, containing addition of few-layered MoS_2_, MoS_2_/Co_2_O_3_/CNT, MoS_2_/Fe_2_O_3_/CNT or MoS_2_/Ni_2_O_3_/CNT, respectively. For each composite three samples containing different amounts of nanomaterials additives were prepared − 0.5 wt%, 1 wt% and 2 wt%, respectively. Following 12 h of drying the nanomaterial ware blended with PLA using twin-screw extruder (Zamak Mercator EHP 2 × 12). For reference a sample of pristine PLA was also extruded.

### Characterization

Morphology of nanomaterials obtained during each stage was analyzed using transmission electron microscopy (TEM) (Tecnai F20, FEI) with 200 kV accelerating voltage. Raman analysis was performed in microscope mode (InVia, Renishaw) with a 785 nm laser in ambient air. Number of layers in few-layered MoS_2_ samples was determined using atomic force microscopy (AFM) (MultiMode 8, Bruker).

For the obtained composites and pristine PLA following analyses were performed. Thermogravimetric analysis (TGA) was performed using thermal analyzer (SDT Q600, TA Instruments) under airflow of 100 mLmin^−1^. In each case individual sample (ca. 5 mg in an alumina crucible) was heated from room temperature to 800 °C at a linear heating rate of 10 °Cmin^−1^. In addition to this, gaseous products from the heating process were analyzed *in situ* with a mass spectrometer (ThermoStar, Pfeiffer Vacuum) coupled with the TGA, under 100 mLmin^−1^ argon flow. Microscale combustion calorimetry (MCC) was employed for measurement of flame retardancy using FAA Micro Calorimeter, from FTT. This allowed for determination of pHHR, heat release capacity (HRC) and total heat release (THR) from 2 mg specimens. Thermal conductivity of the obtained samples was measured using laser flash apparatus (XFA 300, Linseis). Prior to this measurement the samples were spray coated with thin layer of graphite in order to facilitate the absorption of laser at the surface.

## Results and Discussion

**Figure 1:**
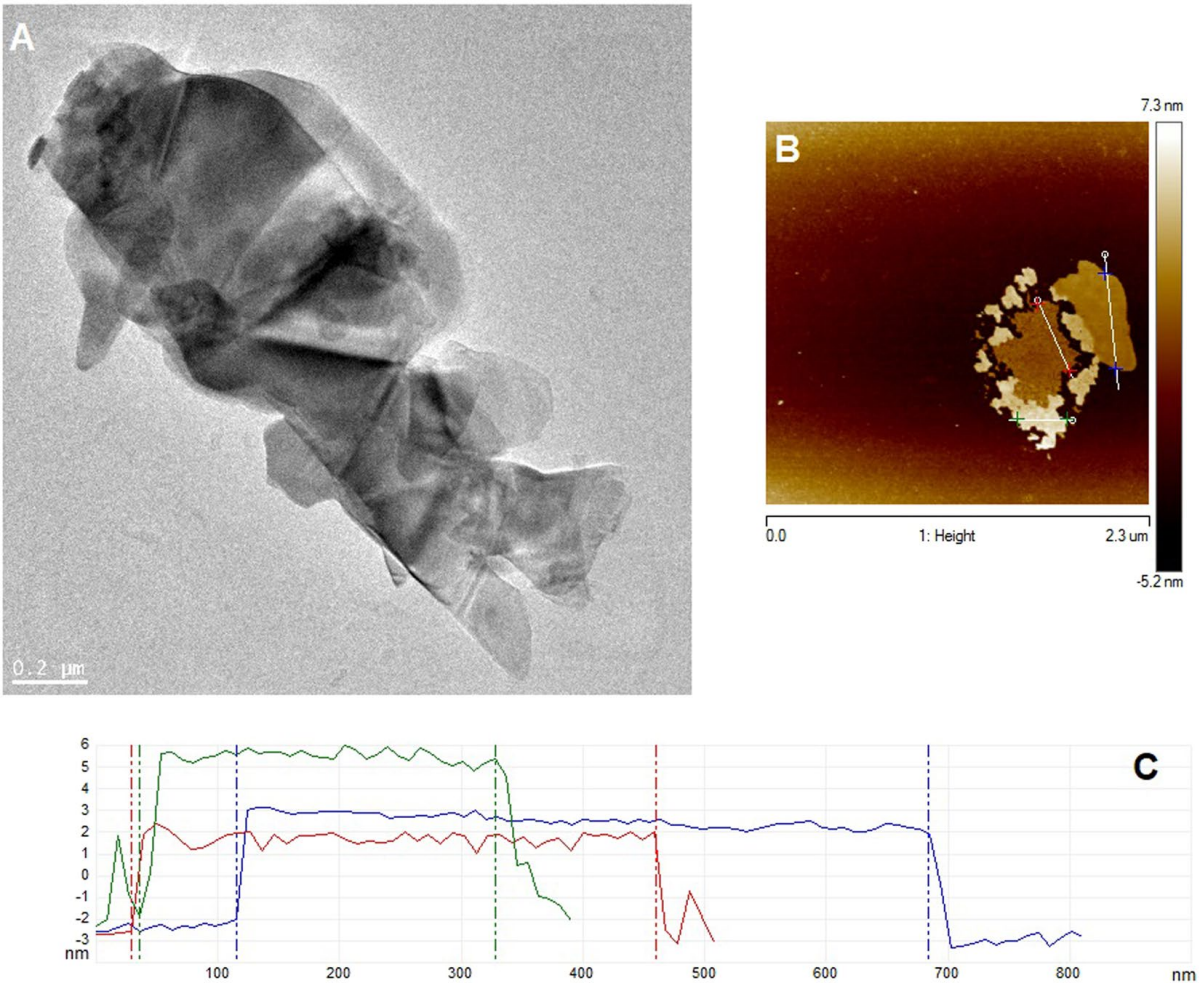
TEM (**A**), AFM (**B**) images of few-layered MoS_2_ and analysis of height profile obtained from AFM (**C**).

Successful preparation of few-layered MoS_2_ samples was confirmed using TEM and AFM (Fig. 1). Number of MoS_2_ layers was determined through analysis of high profile with AFM (Fig. 1C). The flakes were typically 5 nm in height, which corresponded to approximately 7 layers of MoS_2_ (assuming average thickness of single layer ca. 0.7 nm^36^ (Dependence between lateral size and aspect ratio in Supplementary information)). This was verified later with Raman spectroscopy as the intensity of ${E}_{2g}^{1}$ and ${A}_{1g}$ peaks changes between bulk MoS_2_ and few-layered MoS_2_. With the increased number of layers the frequency of ${E}_{2g}^{1}$ (~382 cm^−1^ for bulk MoS_2_) decreases while that of the ${A}_{1g}$ (~407 cm^−1^ for bulk MoS_2_) increases. This effect is caused by the interlayer van der Waals force in MoS_2_ suppressing the atom vibration, which results in higher force constants^36,37^.

**Figure 2:**
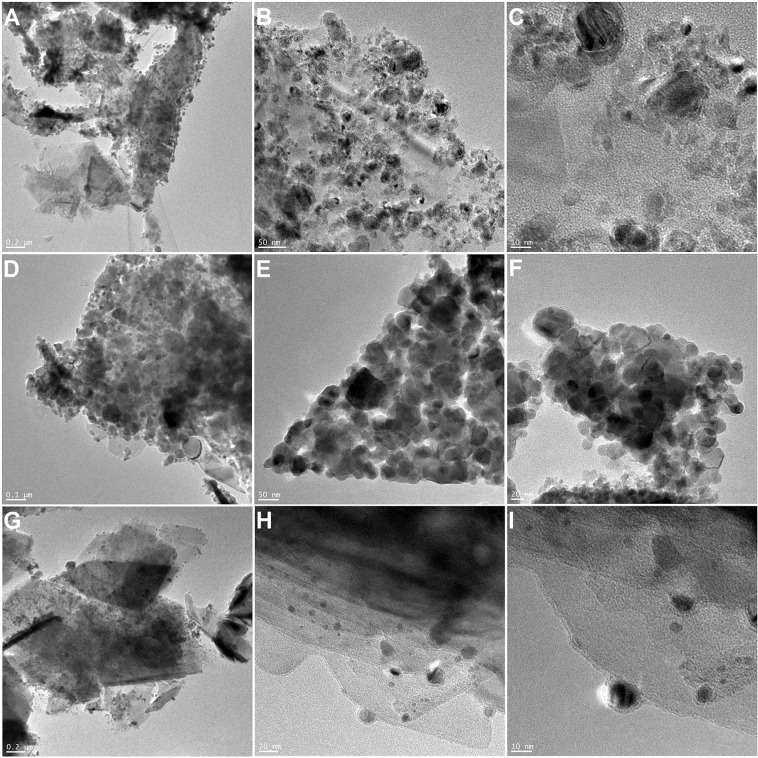
TEM images of MoS_2_/Co_2_O_3_ (**A–C**), MoS_2_/Fe_2_O_3_ (**D–F**) and MoS_2_/Ni_2_O_3_ (**G–I**) nanoparticles.

**Figure 3:**
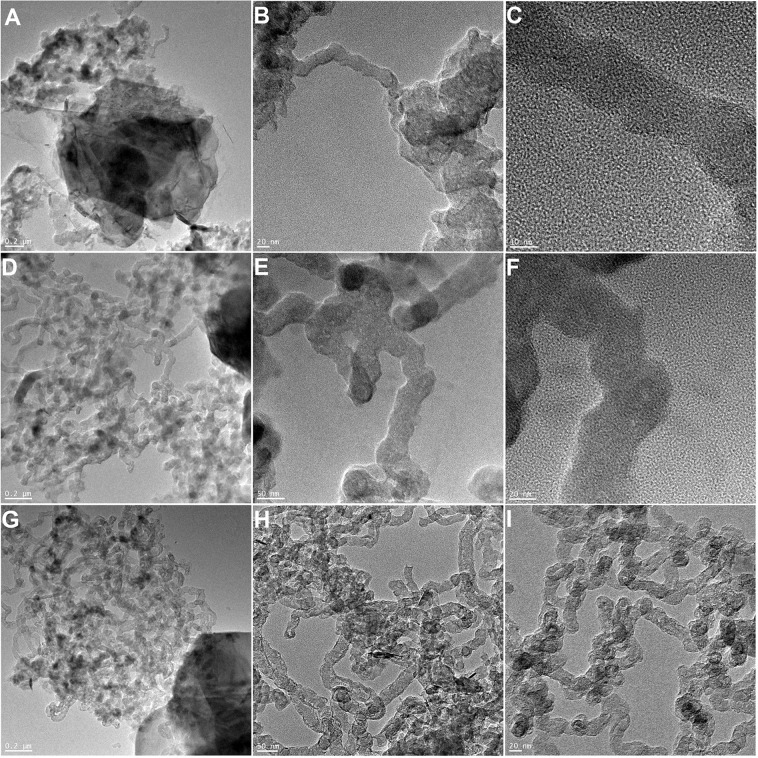
TEM images of MoS_2_/Co_2_O_3_/CNT (**A–C**), MoS_2_/Fe_2_O_3_/CNT (**D–F**) and MoS_2_/Ni_2_O_3_/CNT (**G–I**) nanoparticles.

Dispersion of metal oxide nanoparticles on the surface of MoS_2_ was determined from collected high-resolution TEM images presented in Fig. 2. Metal oxide nanoparticles appeared to be well dispersed on the surface and deposited homogenously. The nanoparticles size was measured and ranged between 5 and 25 nm for all metal oxide nanoparticles. Presence of CNT on samples from the CVD process was confirmed with TEM (Fig. 3). As observed, internal diameter of nanotubes matched the diameter of specific metal oxide nanoparticles. Hallow core and an open end were visible and the surface appeared to be smooth.

### Thermal stability

**Figure 4:**
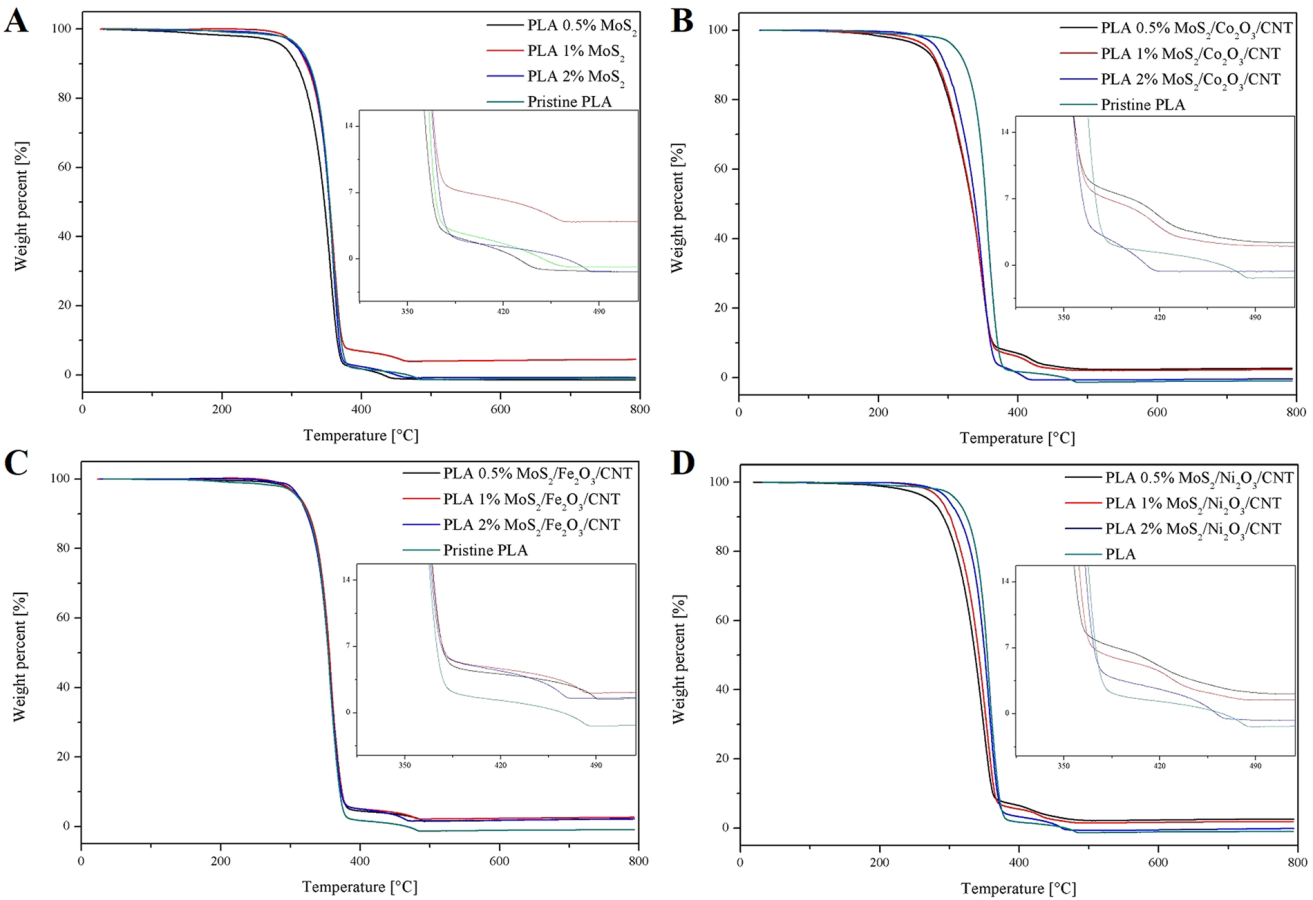
TGA curves of PLA composites in comparison to pristine PLA.

TGA measurements were performed in order to study the influence of MoS_2_ as well as MoS_2_/M_x_O_y_/CNT nanomaterial on the thermal stability of PLA. Figure 4 presents the TGA curves for PLA/MoS_2_ and MoS_2_/M_x_O_y_/CNT composites in comparison to pristine PLA. Based on accumulated data T_10wt%_, T_50wt%_ and T_max_ values (which represent temperatures at which 10%, 50% and maximum weight loss occur) were registered and displayed in Table 1. It can be observed that in case of PLA modified only by the addition of few-layered MoS_2_ (Fig. 4A) at loading of 0.5 wt% thermal decomposition proceeded at a slightly higher rate in comparison to pristine PLA, suggesting accelerated decomposition of polymer matrix as a result of flame retardant (FR) condensed phase action, which affected flow of the polymer^38^. As recorded in this case T_10wt%_ value was lowered and was equal to 306 °C- it was 18 °C below that of a pristine PLA. In addition to this, further reduction in T_50wt%_ and T_max_ values in comparison to pristine PLA was also observed for this sample. In case of samples containing 1 wt% and 2 wt% of loading of few-layered MoS_2_ only T_max_ was strongly affected which suggested that in both cases decomposition of polymer matrix proceeded at a normal rate in initial stages and stopped at a lower temperature. Therefore, it appears that addition of few-layered MoS_2_ nanoparticles has resulted in disruption of heat and gas diffusion. This effect was dependant on the FR load, as well as quality of dispersion in the polymer matrix. Combined with barrier effect, which limited access to fuel, it has caused the combustion process to finish at lower temperature. For samples containing addition of MoS_2_/Co_2_O_3_/CNT (Fig. 4B) a significant decrease in every recorded value was observed. T_10wt%_ values were lowered by up to 40 °C for sample containing 0.5 wt% load of FR. T_50wt%_ values were as much as 20 °C below that of pristine PLA for samples containing 0.5 wt% and 1 wt% FR load. Highest reduction in T_max_ value was observed for sample containing 2 wt% load of FR and was 65 °C below that of pristine PLA. It appears that accelerated decomposition of the PLA matrix was caused by condensed action of the MoS_2_/Co_2_O_3_/CNT^38^. This resulted in pronounced flow of the polymer and its withdrawal from the sphere of influence. Change in melt-flow properties was confirmed during the preparation of samples for thermal conductivity testing. Composite samples needed to be preheated to glass transition temperature (about 150 °C) to form tablets using a table press. In case of PLA containing addition of MoS_2_/Co_2_O_3_/CNT the glass transition temperature appeared to be significantly lower (equal to about 110 °C) in comparison to another samples. In literature it was reported that poly(lactic acid) and poly(methyl methacrylate) (PMMA) blends containing addition of isopropylated triaryl phosphate ester flame retardant displayed accelerated breakdown of polymer during the TGA testing, combined with accelerated melt dripping during the UL-94 rating testing, favouring the melt-flow drip mode of extinguishment^3^. As result of this, PLA/PMMA/FR blends have achieved V-0 classification in the UL-94 test^3^. In case of MoS_2_/Fe_2_O_3_/CNT (Fig. 4C) only a significant reduction in T_max_ value was observed, which was highest (14 °C) for sample containing 2 wt% of nanomaterial, while the largest amount of charred reside was observed with sample containing 1 wt%. Recorded T_10wt%_ and T_50wt%_ values remained within the range of temperatures observed for pristine PLA. The composite samples containing addition of MoS_2_/Ni_2_O_3_/CNT (Fig. 4D) placed between those derived from Co_2_O_3_ and Fe_2_O_3_ nanoparticles – at lower FR loads polymer decomposition was accelerated but the stability of matrix increased with wt% of FR. In summary, obtained results suggest improved fire resistant performance due to disruption of heat and gas transfer within the composites, as well as barrier effect introduced through the addition of MoS_2_ and CNT.

**Table 1:** Summary of TGA results for PLA and PLA composites.

	FR load [wt%]	T_10wt%_ [°C]	T_50wt%_ [°C]	T_max_ [°C]
PLA	—	324	354	485
	0.5	306	346	445
PLA MoS_2_	1	320	351	459
	2	322	353	465
	0.5	284	334	496
PLA MoS_2_/Co_2_O_3_/CNT	1	286	334	488
	2	295	339	420
	0.5	326	354	491
PLA MoS_2_/Fe_2_O_3_/CNT	1	326	356	483
	2	323	354	469
	0.5	291	337	493
PLA MoS_2_/Ni_2_O_3_/CNT	1	301	342	480
	2	314	351	468

**Figure 5:**
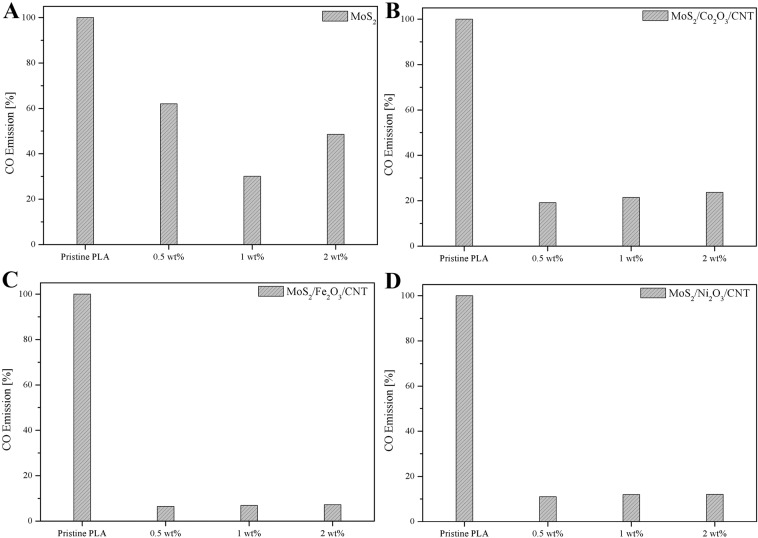
CO emission of PLA composites in comparison to pristine PLA.

CO content analysis performed with use of mass spectrometry during the TGA pyrolysis cycle in argon atmosphere revealed promising results. In each case the amount of CO released was lower in comparison to that observed for pristine PLA (Fig. 5). For PLA modified only with addition of few-layered MoS_2_ (Fig. 5A) the reduction in CO release ranged from 38 up to 70%, with the highest value recorded for sample containing 1 wt% of nanomaterials which also corresponded to the largest recorded charred residue value. Previously we observed that introduction of few-layered MoS_2_ into the poly(vinyl alcohol) (PVA) composite resulted in significant decrease (up to 94%) in permeability of hydrogen gas in comparison to pristine PVA^39^. This effect was attributed to the incorporation of impermeable MoS_2_ nanosheets with high aspect ratio. As a result a high number of tortuous pathways was created which restricted the diffusion of penetrants, such as H_2_ or CO^39,40^. Reduction of CO emission during pyrolysis was further enhanced in case of MoS_2_/M_x_O_y_/CNT composites. Highest reduction of CO emission in respect to pristine PLA was observed for all samples containing MoS_2_/Fe_2_O_3_/CNT (Fig. 5C), and it was always above 90%. This was attributed to a well developed and thermally stable char barrier, which protected the molten zone from the burning zone, as reported by Attia *et al*.^41^ in case of polystyrene composites containing addition of MWCNT.

### Flammability studies

**Figure 6:**
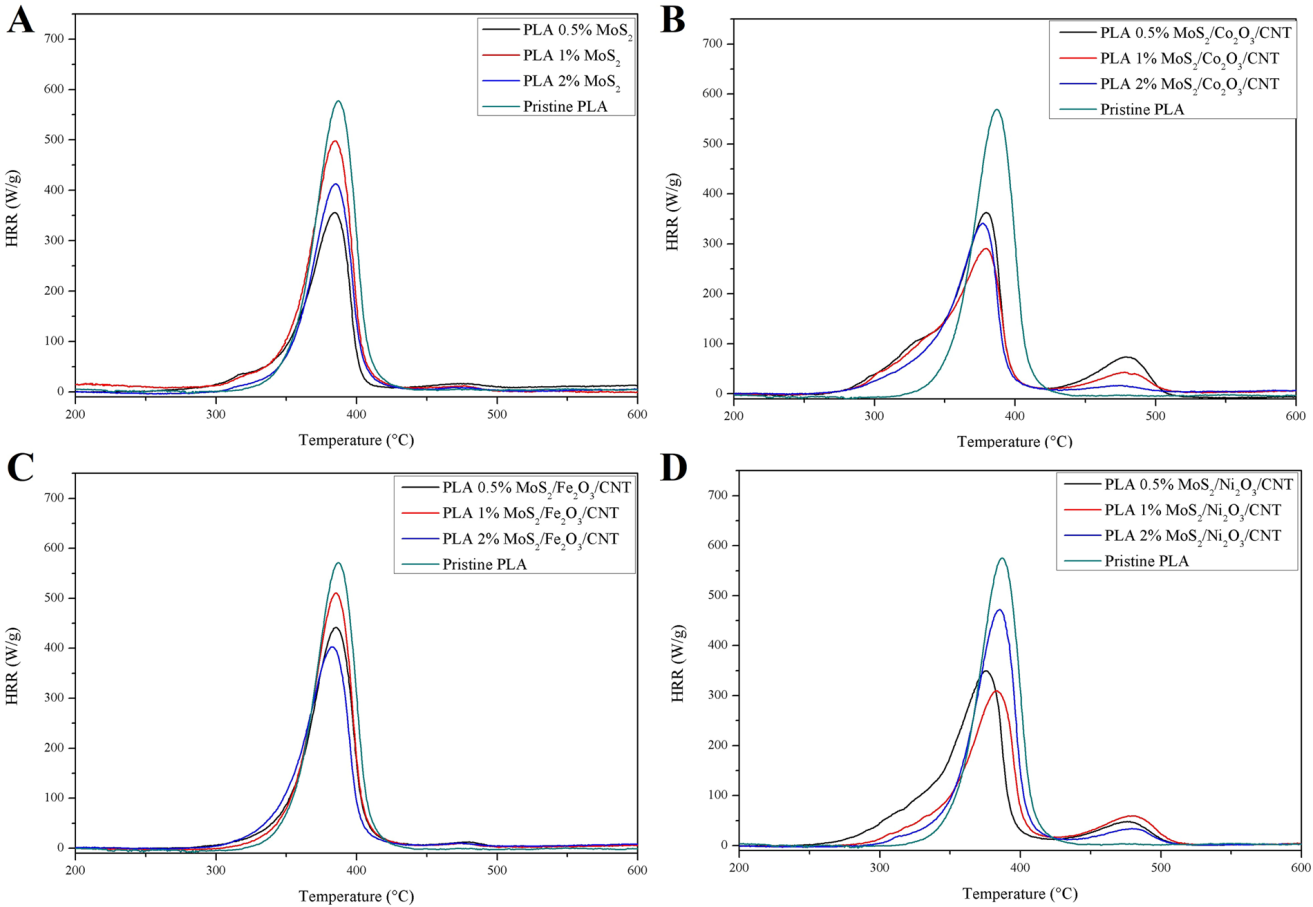
Examples of HRR curves obtained for pristine PLA and PLA composites.

Fire performance testing was conducted using MCC, which allowed for laboratory evaluation of fire resistant properties of composites. Examples of heat release rate curves of prepared composites were presented in Fig. 6. Average values for key parameters - pHRR, HRC and THR were calculated from three measurements for each sample and presented in Table 2. All of these values were lowered for samples containing the addition of FR in comparison to pristine PLA. The samples containing an addition of MoS_2_ (Fig. 6A) showed satisfying FR performance with significant decrease of pHRR and THR values in comparison to pristine PLA. Therefore, it was evident that the addition of MoS_2_ into the PLA matrix introduced the barrier effect which effectively impeded oxygen filtration, heat and mass transmission and the release of combustible gases produced during the combustion process, as previously reported for PVA, polystyrene (PS) and PMMA^11,42,43^. Formation of this barrier prevented underlying material from further combustion. For composite samples containing addition of MoS_2_/M_x_O_y_/CNT FR best results were observed for 2 wt% load. This effect could be attributed to strong barrier effect due to formation of an enhanced char layer thanks to the presence of CNT^21,44^. In case of samples containing addition of MoS_2_/Co_2_O_3_/CNT (Fig. 6B) and MoS_2_/Ni_2_O_3_/CNT (Fig. 6D) there was a visible shoulder within the temperature range of 250–350 °C. This suggested occurrence of an overlapping stage, preceding main combustion of PLA. This may be attributed to the release of FR additive via vaporisation or cleavage. In addition, a secondary peak was also observed for those samples within the temperature range of 425–515 °C which could be attributed to the increase in volume of sample due to intumescence. The intensity of this peak decreased with increase in FR loading, which suggests that rigidity of the barrier was directly tied to the FR contents and it is optimal at 2 wt%. While both of the effects could be also observed for samples containing addition of MoS_2_ as well as MoS_2_/Fe_2_O_3_/CNT (Fig. 6C), the size of the before mentioned shoulder as well as intensity of the secondary peak were much lower.

**Table 2:** MCC combustion data of PLA and PLA composites.

	FR load [wt%]	HRC [Jg^−1^K^−1^]	pHRR [Wg^−1^]	THR [kJg^−1^]
PLA	—	703.6	558.3	22.1
	0.5	555.2	395.9	16.6
PLA MoS_2_	1	618.9	476.3	18.8
	2	602.6	468.6	17.9
	0.5	560.2	337.1	21.6
PLA MoS_2_/Co_2_O_3_/CNT	1	571.3	332.1	20.4
	2	565.4	318.7	19.1
	0.5	621.6	483.7	19.6
PLA MoS_2_/Fe_2_O_3_/CNT	1	607.2	471.6	18.7
	2	595.9	449.5	17.9
	0.5	550.2	375.2	21.1
PLA MoS_2_/Ni_2_O_3_/CNT	1	548.2	363.3	20.2
	2	543.3	330.8	19.8

### Thermal conductivity

In order to verify thermal conductivity of obtained composites samples with a diameter of 12.7 mm were prepared using tablet press. For each composite a batch of 3 tablets were prepared. Thickness of each tablet was measured and recorded. In order to facilitate laser adsorption at the surface all tablets were covered with a thin layer of graphite prior to measurement. Tests were carried out at room temperature, which was measured at 25.4 °C. Obtained data (average out of 3 tablets for each composite) was presented in Table 3. In case of samples prepared with addition of only few-layered MoS_2_ highest level of thermal conductivity – 0.502 Wm^−1^K^−1^ was observed for sample containing addition of 2 wt% of FR and it was 54% higher than that of a pristine PLA sample. Similar effects were reported in literature for epoxy samples containing addition of nanomaterial such as graphene oxide or graphene nanosheets^45^. For samples containing CNT based structures thermal conductivity increased with increase in loading, resulting in up to 0.538 Wm^−1^K^−1^ or a 65% increase over pristine PLA in case of a sample containing 2 wt% of MoS_2_/Fe_2_O_3_/CNT. It is however worth noting that even in case of 0.5 wt% of MoS_2_/Ni_2_O_3_/CNT a 50.9% increase in thermal conductivity was observed. Kashiwagi *et al*. also observed similar increase in thermal conductivity in case of polypropylene composites containing addition of carbon nanotubes which suggests that further enhancement of thermal conductivity of CNT based composites could be attributed to their presence^46^.

**Table 3:** Thermal conductivity of PLA and PLA composites.

	FR load [wt%]	Thermal conductivity [W m^−1^K^−1^]	Increase from pristine PLA [%]
PLA	—	0.326	
	0.5	0.365	12.0
PLA MoS_2_	1	0.421	29.1
	2	0.502	54.0
	0.5	0.341	4.6
PLA MoS_2_/Co_2_O_3_/CNT	1	0.392	20.2
	2	0.465	42.6
	0.5	0.466	42.9
PLA MoS_2_/Fe_2_O_3_/CNT	1	0.487	49.4
	2	0.538	65.0
	0.5	0.492	50.9
PLA MoS_2_/Ni_2_O_3_/CNT	1	0.500	53.4
	2	0.510	56.4

## Conclusions

In this work, in order to improve fire performance of PLA, few-layered MoS_2_ and MoS_2_/M_x_O_y_/CNT nanomaterials were prepared and introduced into the PLA composites through twin-screw extrusion blending process. TGA analysis revealed 40 °C and 65 °C decrease of T_max_ value for samples containing 0.5 wt% of few-layered MoS_2_ and 2 wt% of MoS_2_/Co_2_O_3_/CNT, respectively. Thanks to the addition of few-layered MoS_2_ a formation of charred residue layer was observed which allowed for up to nearly 70% decrease in CO emission during burning in case of a sample containing 1 wt% of MoS_2_. Over 90% of reduction in CO emission was achieved through introduction of MoS_2_/Fe_2_O_3_/CNT into the PLA matrix. MCC analysis showed best performance of few-layered MoS_2_ and MoS_2_/Fe_2_O_3_/CNT composites, while MoS_2_/Co_2_O_3_/CNT and MoS_2_/Ni_2_O_3_/CNT composites displayed strong effects of secondary stage preceding the main combustion of PLA as well as instability of barrier at 0.5 wt% and 1 wt% of FR. Thermal conductivity of all of the composites was increased in comparison to pristine PLA, up to 65% in case of a composite containing addition of 2 wt% of MoS_2_/Fe_2_O_3_/CNT due to the presence of CNT. In conclusion, introduction of few-layered MoS_2_ and CNT functionalized MoS_2_ nanomaterials shows good potential for reduction of fire hazard of the PLA, which could prove beneficial for academic research and practical applications.

## Supplementary information

Suplemetary for manuscript.

## Data Availability

The datasets used and/or analysed during the current study are available from the corresponding author on reasonable request.

## References

[1] Bocz K (2015). Flame retarded self-reinforced poly(lactic acid) composites of outstanding impact resistance. Compos Part A Appl Sci Manuf.

[2] Fox DM, Lee J, Citro CJ, Novy M (2013). Flame retarded poly(lactic acid) using POSS-modified cellulose. Polym Degrad Stab..

[3] Teoh EL, Mariatti M, Chow WS (2013). Thermal and Flame Resistant Properties of Poly (Lactic Acid)/Poly (Methyl Methacrylate) Blends Containing Halogen-free Flame Retardant. Procedia Chem.

[4] Zhao X, Gao S, Liu GA (2016). THEIC-based polyphosphate melamine intumescent flame retardant and its flame retardancy properties for polylactide. J Anal Appl Pyrolysis.

[5] Liu XQ, Wang DY, Wang XL, Chen L, Wang YZ (2013). Synthesis of functionalized α-zirconium phosphate modified with intumescent flame retardant and its application in poly(lactic acid). Polym Degrad Stab.

[6] Liao F (2015). A novel efficient polymeric flame retardant for poly (lactic acid) (PLA): Synthesis and its effects on flame retardancy and crystallization of PLA. Polym Degrad Stab.

[7] Costes L (2014). Phosphorus and nitrogen derivatization as efficient route for improvement of lignin flame retardant action in PLA. Eur Polym J.

[8] Fontaine G, Bourbigot S (2009). Intumescent polylactide: A nonflammable material. J Appl Polym Sci.

[9] Qian Y (2013). Aluminated mesoporous silica as novel high-effective flame retardant in polylactide. Compos Sci Technol.

[10] Zhang S (2013). Intercalation of phosphotungstic acid into layered double hydroxides by reconstruction method and its application in intumescent flame retardant poly (lactic acid) composites. Polym Degrad Stab.

[11] Zhou K, Liu J, Zeng W, Hu Y, Gui Z (2013). *In situ* synthesis, morphology, and fundamental properties of polymer/MoS2 nanocomposites. Compos Sci Technol.

[12] Wang D, Xing W, Song L, Hu Y (2016). Space-Confined Growth of Defect-Rich Molybdenum Disulfide Nanosheets Within Graphene: Application in the Removal of Smoke Particles and Toxic Volatiles. ACS Appl Mater Interfaces.

[13] Zhou K (2013). Comparative study on the thermal stability, flame retardancy and smoke suppression properties of polystyrene composites containing molybdenum disulfide and graphene. RSC Adv.

[14] Guo Y (2017). Capitalizing on the molybdenum disulfide/graphene synergy to produce mechanical enhanced flame retardant ethylene-vinyl acetate composites with low aluminum hydroxide loading. Polym Degrad Stab.

[15] Hong N (2018). Facile preparation of graphene supported Co3O4 and NiO for reducing fire hazards of polyamide 6 composites. Mater Chem Phys.

[16] Wang X (2018). The effect of metal oxide decorated graphene hybrids on the improved thermal stability and the reduced smoke toxicity in epoxy resins. Chem Eng J.

[17] Wang X, Kalali EN, Wan J-TT, Wang DYY (2017). Carbon-family materials for flame retardant polymeric materials. Prog Polym Sci.

[18] Kashiwagi T (2005). Flammability properties of polymer nanocomposites with single-walled carbon nanotubes: Effects of nanotube dispersion and concentration. Polymer (Guildf).

[19] Hapuarachchi TD, Peijs T (2016). Multiwalled carbon nanotubes and sepiolite nanoclays as flame retardants for polylactide and its natural fibre reinforced composites. Compos Part A Appl Sci Manuf.

[20] Kashiwagi T (2002). Thermal degradation and flammability properties of poly(propylene)/carbon nanotube composites. Macromol Rapid Commun.

[21] Verdejo R (2008). Carbon nanotubes provide self-extinguishing grade to silicone-based foams. J Mater Chem.

[22] Zúñiga C (2014). Convenient and solventless preparation of pure carbon nanotube/polybenzoxazine nanocomposites with low percolation threshold and improved thermal and fire properties. J Mater Chem A.

[23] Wu Q, Zhu W, Zhang C, Liang Z, Wang B (2010). Study of fire retardant behavior of carbon nanotube membranes and carbon nanofiber paper in carbon fiber reinforced epoxy composites. Carbon N Y.

[24] Wang L, Jiang PK (2011). Thermal and flame retardant properties of ethylene-vinyl acetate copolymer/modified multiwalled carbon nanotube composites. J Appl Polym Sci.

[25] Kuan CF (2010). Flame retardance and thermal stability of carbon nanotube epoxy composite prepared from sol–gel method. J Phys Chem Solids.

[26] He Q (2013). Flame-Retardant Polypropylene/Multiwall Carbon Nanotube Nanocomposites: Effects of Surface Functionalization and Surfactant Molecular Weight. Macromol Chem Phys.

[27] Xu G (2013). Functionalized carbon nanotubes with oligomeric intumescent flame retardant for reducing the agglomeration and flammability of poly(ethylene vinyl acetate) nanocomposites. Polym Compos.

[28] Peeterbroeck S (2007). Mechanical Properties and Flame-Retardant Behavior of Ethylene Vinyl Acetate/High-Density Polyethylene Coated Carbon Nanotube Nanocomposites. Adv Funct Mater.

[29] Ma HY, Tong LF, Xu ZB, Fang ZP (2008). Functionalizing Carbon Nanotubes by Grafting on Intumescent Flame Retardant: Nanocomposite Synthesis, Morphology, Rheology, and Flammability. Adv Funct Mater.

[30] Song P, Xu L, Guo Z, Zhang Y, Fang Z (2008). Flame-retardant-wrapped carbon nanotubes for simultaneously improving the flame retardancy and mechanical properties of polypropylene. J Mater Chem.

[31] Pal K, Kang DJ, Zhang ZX, Kim JK (2016). Synergistic Effects of Zirconia-Coated Carbon Nanotube on Crystalline Structure of Polyvinylidene Fluoride Nanocomposites: Electrical Properties and Flame-Retardant Behavior. Langmuir.

[32] Zhang T, Du Z, Zou W, Li H, Zhang C (2012). The flame retardancy of blob-like multi-walled carbon nanotubes/silica nanospheres hybrids in poly (methyl methacrylate). Polym Degrad Stab.

[33] Du B, Fang Z (2014). The preparation of layered double hydroxide wrapped carbon nanotubes and their application as a flame retardant for polypropylene. Nanotechnology.

[34] Gao F, Beyer G, Yuan Q (2005). A mechanistic study of fire retardancy of carbon nanotube/ethylene vinyl acetate copolymers and their clay composites. Polym Degrad Stab.

[35] Ma H, Tong L, Xu Z, Fang Z (2007). Synergistic effect of carbon nanotube and clay for improving the flame retardancy of ABS resin. Nanotechnology.

[36] Wang X, Feng H, Wu Y, Jiao L (2013). Controlled synthesis of highly crystalline MoS2 flakes by chemical vapor deposition. J Am Chem Soc.

[37] Placidi M (2015). Multiwavelength excitation Raman scattering analysis of bulk and two-dimensional MoS2: Vibrational properties of atomically thin MoS2 layers. 2D Mater.

[38] Bourbigot S, Fontaine G (2010). Flame retardancy of polylactide: An overview. Polym Chem.

[39] Thakur S, Bandyopadhyay P, Kim SH, Kim NH, Lee JH (2018). Enhanced physical properties of two dimensional MoS2/poly(vinyl alcohol) nanocomposites. Compos Part A Appl Sci Manuf.

[40] Huang HD (2012). High barrier graphene oxide nanosheet/poly(vinyl alcohol) nanocomposite films. J Memb Sci.

[41] Attia NF, Afifi HA, Hassan MA (2015). Synergistic Study of Carbon Nanotubes, Rice Husk Ash and Flame Retardant Materials on the Flammability of Polystyrene Nanocomposites. Mater. Today Proc..

[42] Zhou K, Gao R, Gui Z, Hu Y (2015). The effective reinforcements of functionalized MoS2 nanosheets in polymer hybrid composites by sol-gel technique. Compos Part A Appl Sci Manuf.

[43] Zhou K, Tang G, Gao R, Guo H (2018). Constructing hierarchical polymer@MoS2 core-shell structures for regulating thermal and fire safety properties of polystyrene nanocomposites. Compos Part A Appl Sci Manuf.

[44] Aschberger K, Campia I, Pesudo LQ, Radovnikovic A, Reina V (2017). Chemical alternatives assessment of different flame retardants – A case study including multi-walled carbon nanotubes as synergist. Pergamon.

[45] Liu S, Chevali VS, Xu Z, Hui D, Wang H (2018). A review of extending performance of epoxy resins using carbon nanomaterials. Compos Part B Eng.

[46] Kashiwagi T (2004). Thermal and flammability properties of polypropylene / carbon nanotube nanocomposites. Polymer (Guildf).

