# Supplementary material for: Enhanced thermal properties of poly(lactic acid)/MoS2/carbon nanotubes composites
Source: Sci Rep. 2020 Jan 20;10:740. doi: 10.1038/s41598-020-57708-1 (PMC6971244; doi:10.1038/s41598-020-57708-1)
Supplement: Supplementary file 1 — Suplemetary for manuscript. [file 41598_2020_57708_MOESM1_ESM.docx]

**Supplementary materials
for
Enhanced thermal properties of poly(lactic acid)/MoS_2_/carbon nanotubes composites**

Piotr Homa, Karolina Wenelska, Ewa Mijowska*

West Pomeranian University of Technology, Szczecin

Faculty of Chemical Technology and Engineering,

Nanomaterials Physicochemistry Department,

Piastów Ave. 42, 71-065 Szczecin, Poland

*Corresponding author, e-mail: [ewa.mijowska@zut.edu.pl](mailto:ewa.mijowska@zut.edu.pl)


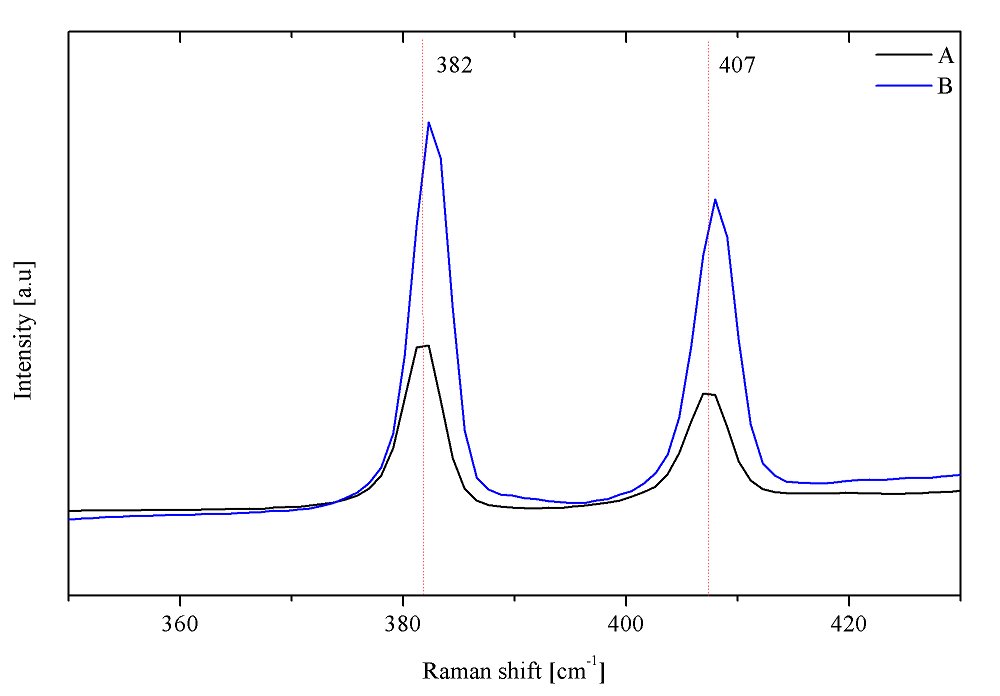


**Figure S1.** Raman spectroscopy of A – bulk MoS_2_ B – few-layered MoS_2._


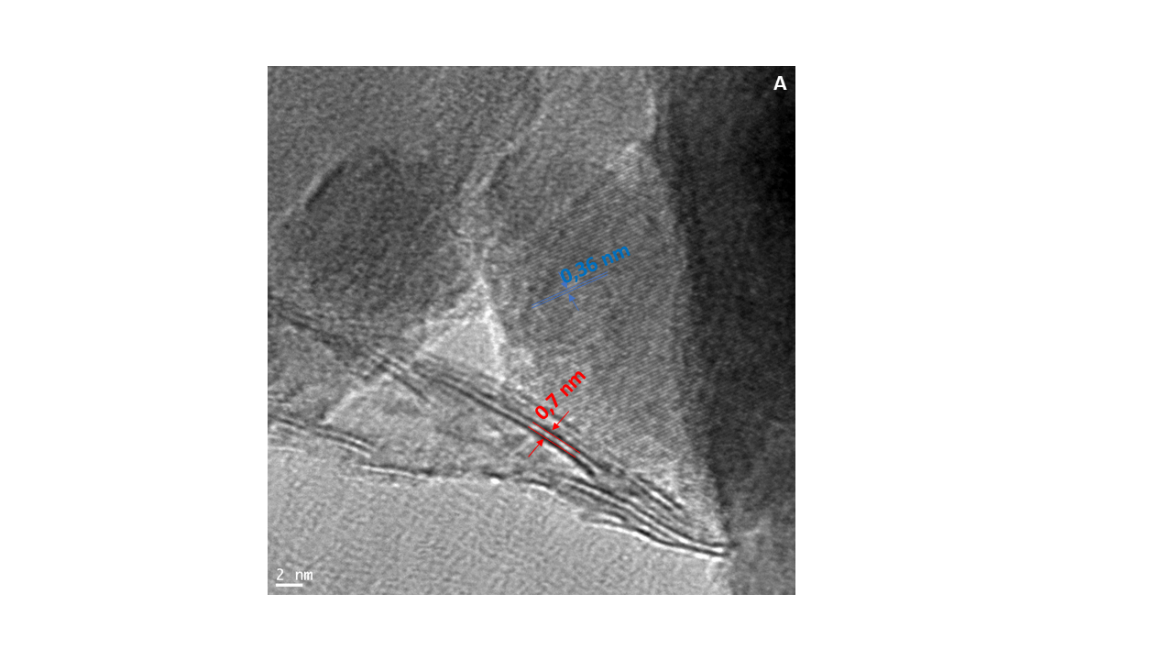


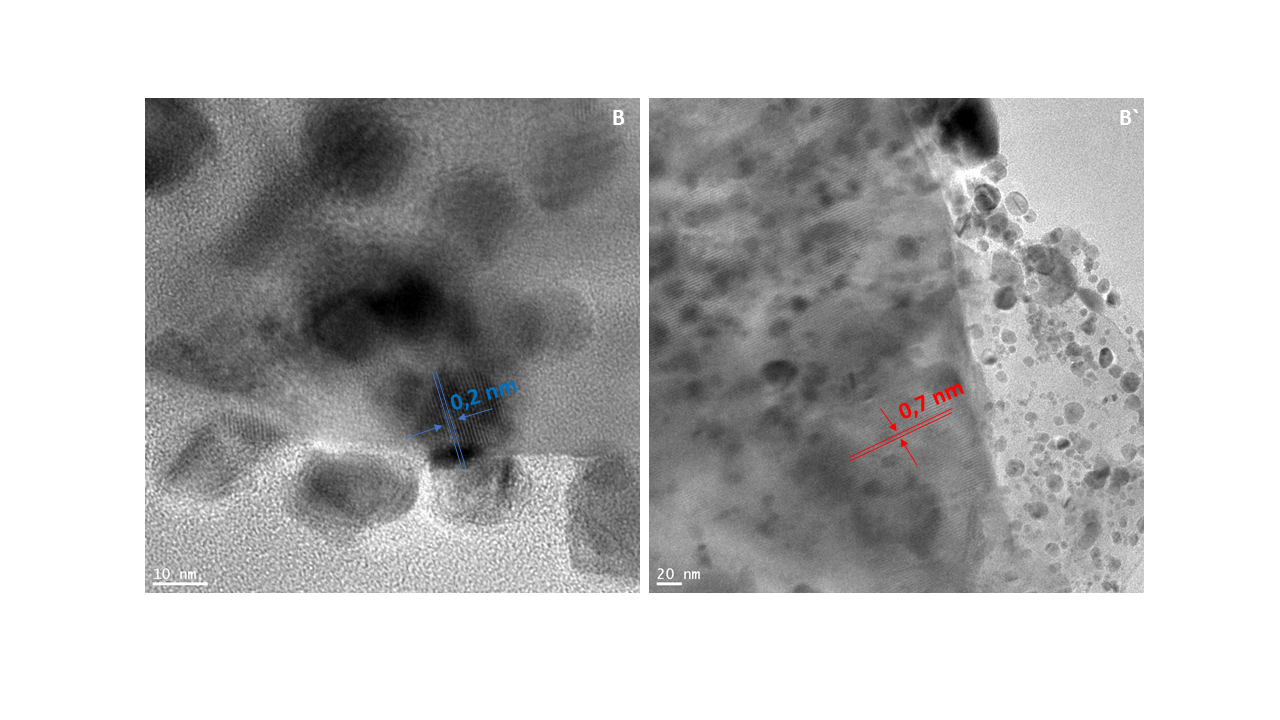


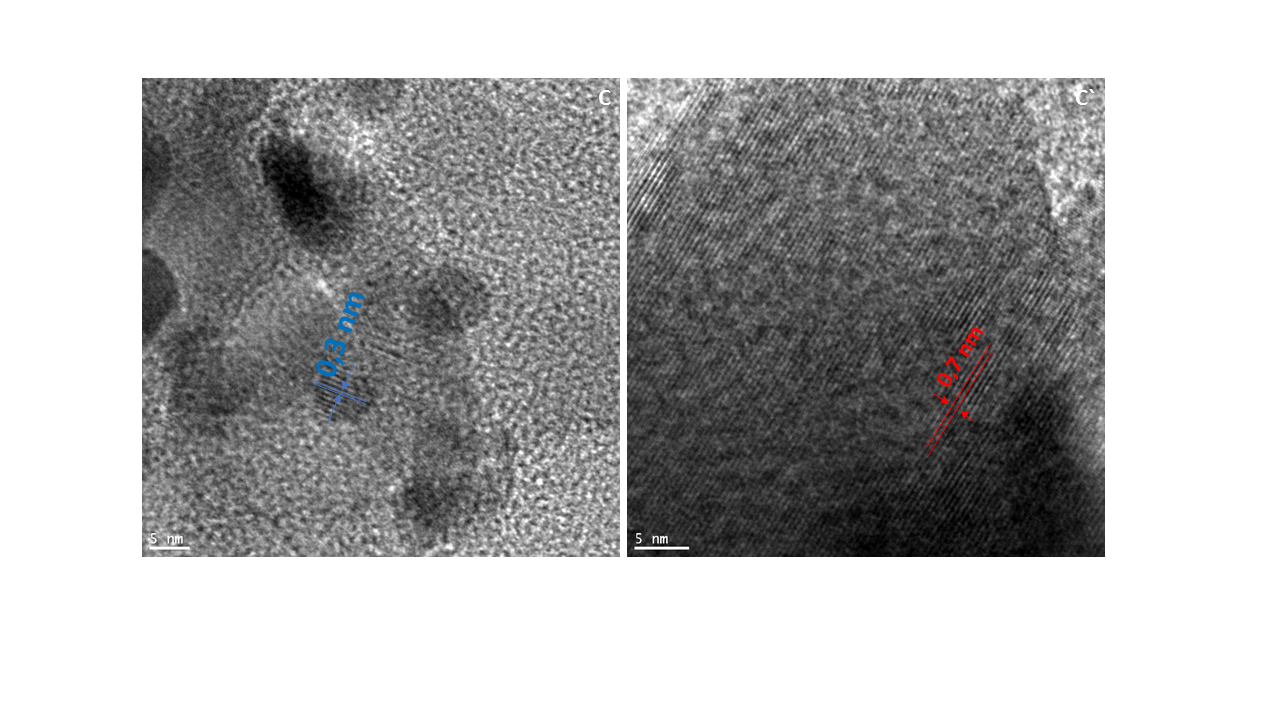

**Figure S2.** TEM images of (A) MoS_2_/Fe_2_O_3_, (B, B`) MoS_2_/Ni_2_O_3_, (C, C`) MoS_2_/Co_2_O_3._

**Figure S3.** XRD pattern of few-layered MoS_2_.

**Figure S4.** Lateral size distributions of MoS_2_.


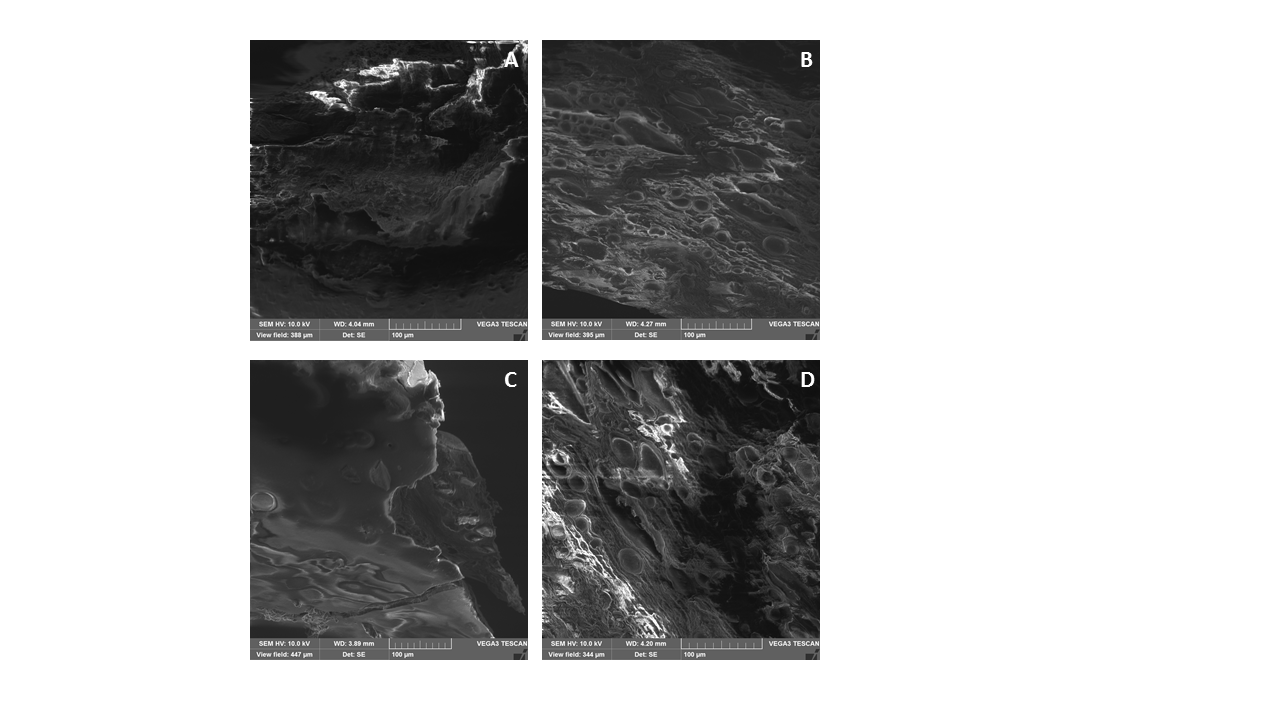


**Figure S5.** SEM images of (A) MoS_2,_ (B) MoS_2_/Fe_2_O_3_, (C) MoS_2_/Ni_2_O_3_, (D) MoS_2_/Co_2_O_3._


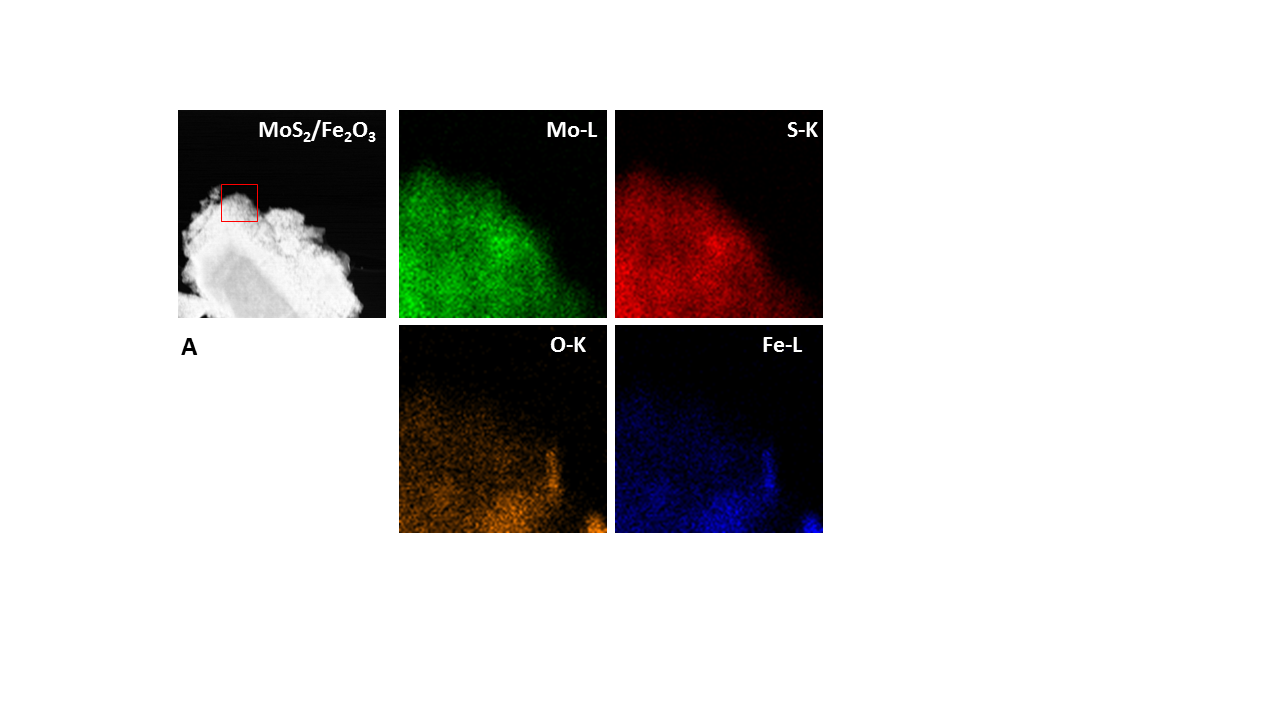


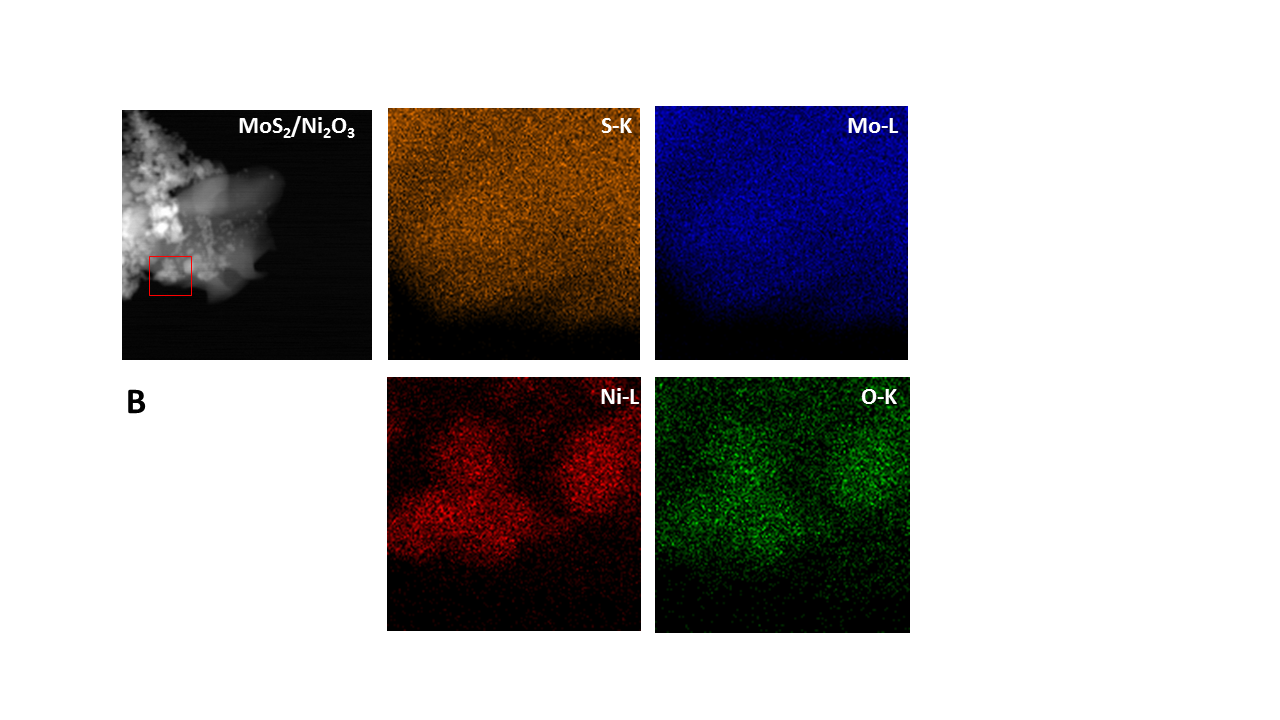


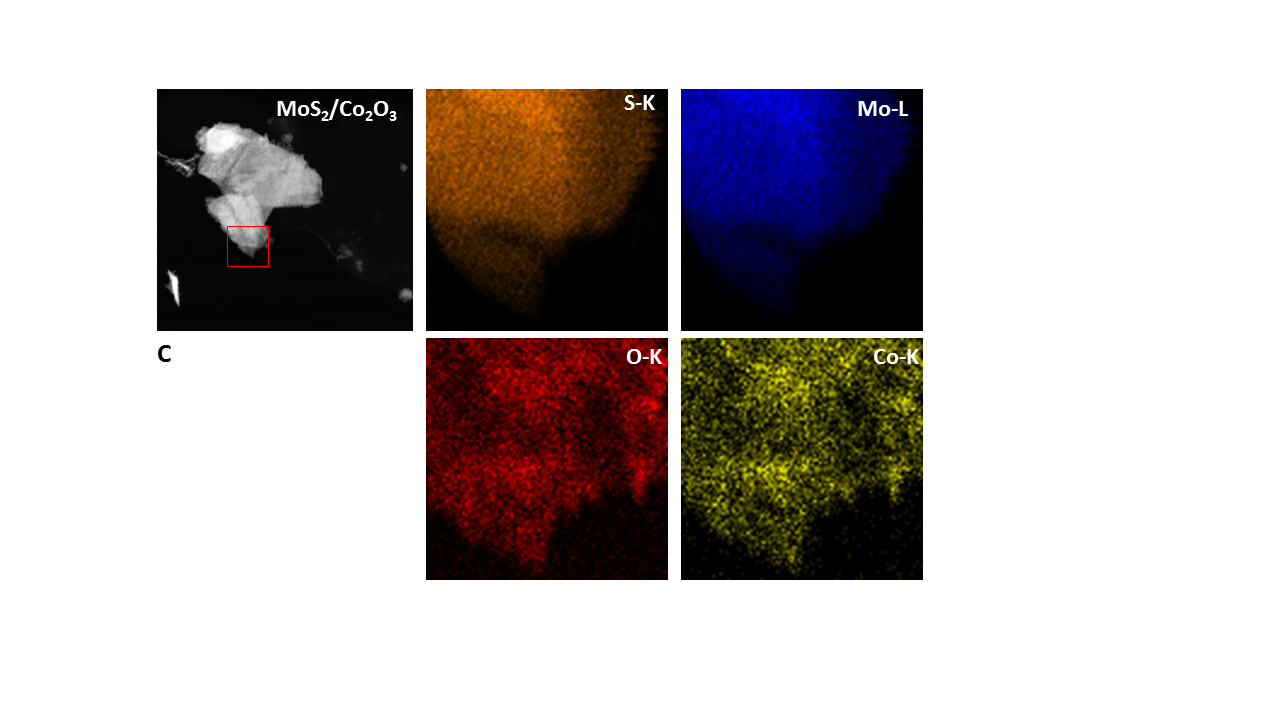


**Figure S6.** EDS mapping of compositions of (A) MoS_2_/Fe_2_O_3_, (B) MoS_2_/Ni_2_O_3_, (C) MoS_2_/Co_2_O_3._

**Figure S8**. TGA profile of (A) MoS_2,_ (B) MoS_2_/Fe_2_O_3_, MoS_2_/Ni_2_O_3_, MoS_2_/Co_2_O_3_ and (C) MoS_2_/Fe_2_O_3_/CNT and MoS_2_/Ni_2_O_3_/CNT, MoS_2_/Co_2_O_3_/CNT.

**Table S1.** The ratios of individual components in MoS_2_/M_x_O_y_/CNT.

|  | **MoS_2_/Fe_2_O_3_/CNT** | **MoS_2_/Ni_2_O_3_/CNT** | **MoS_2_/Co_2_O_3_/CNT** |
| --- | --- | --- | --- |
| **MxOy [%]** | 60 | 48 | 37 |
| **CNT [%]** | 14 | 9 | 5 |
| **MoS_2_ [%]** | 26 | 43 | 58 |


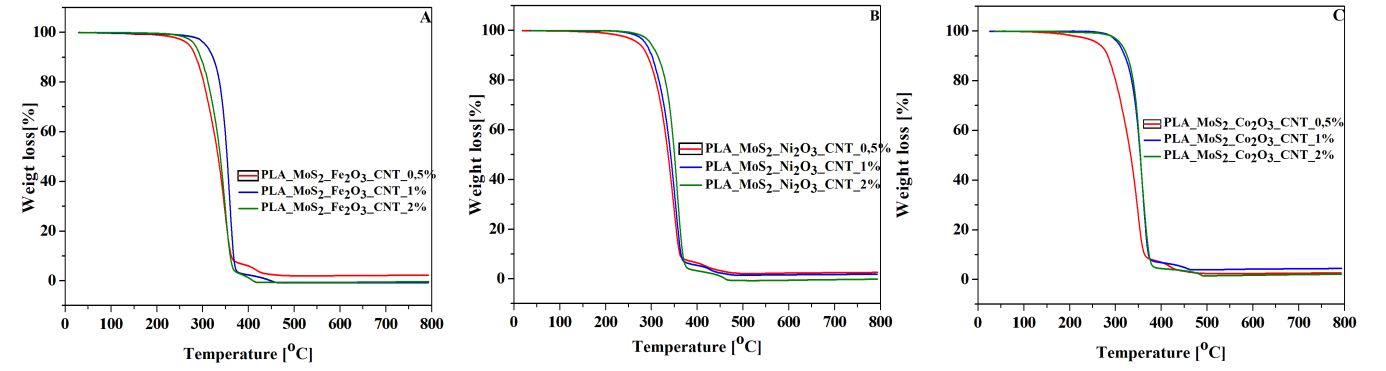


**Figure S9.** TGA profile of simple mixtures of MoS_2_, M_x_O_y_, and CNT (A)MoS_2_, Fe_2_O_3_, CNT, (B) MoS_2_, Ni_2_O_3_, CNT (C) MoS_2_, Co_2_O_3,_ CNT.


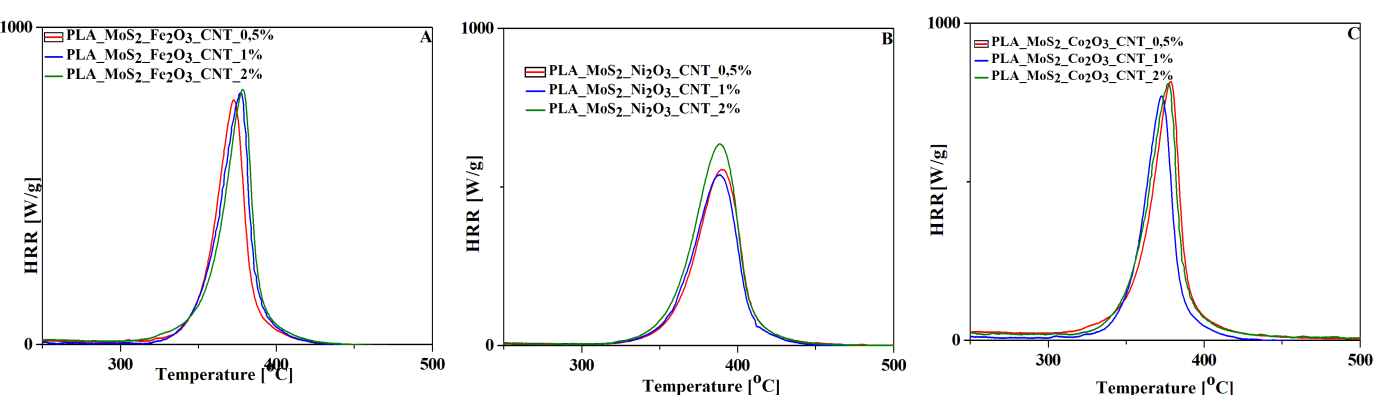


**Figure S10**. HRR curves of simple mixtures of MoS_2_, M_x_O_y_, and CNT (A)MoS_2_, Fe_2_O_3_, CNT, (B) MoS_2_, Ni_2_O_3_, CNT (C) MoS_2_, Co_2_O_3,_ CNT.

**Figure S11.** Dependence between lateral size and aspect ratio.

**Figure S12.** XRD patterns of (a) MoS_2_ (b) MoS_2_/Co_2_O_3,_ (c) MoS_2_/Fe_2_O_3_, (d) MoS_2_/Ni_2_O_3._
